# Improvement in Left Ventricular Function with Intracoronary Mesenchymal Stem Cell Therapy in a Patient with Anterior Wall ST-Segment Elevation Myocardial Infarction

**DOI:** 10.1007/s10557-018-6804-z

**Published:** 2018-06-28

**Authors:** Su Hyun Kim, Jang Hyun Cho, Yoon Ho Lee, Ji Hye Lee, Soo Sung Kim, Mi Yang Kim, Min Gu Lee, Won Yu Kang, Kyung Sim Lee, Young Keun Ahn, Myung Ho Jeong, Hyun Soo Kim

**Affiliations:** 1 Cardiovascular Intervention, St. Carollo Hospital, Suncheon-si, Jeollanam-do South Korea; 2 Department of Internal Medicine, St. Carollo Hospital, Suncheon-si, Jeollanam-do South Korea; 3 Department of Nursing, Gwangyang Health Science University, Gwangyang-si, Jeollanam-do South Korea; 4 Cardiovascular Center, Department of Cardiology, Chonnam National University Hospital, Gwangju, South Korea; 5 Dr. Kim’s Stem Cell Clinic, Seoul, South Korea

**Keywords:** Acute myocardial infarction, Heart failure, Mesenchymal stem cell

## Abstract

**Background/Aims:**

The progression and development of congestive heart failure is still considered a large problem despite the existence of revascularization therapies and optimal, state-of-the-art medical services. An acute myocardial infarction (AMI) is a major cause of congestive heart failure, so researchers are investigating techniques to complement primary percutaneous coronary intervention (PCI) or thrombolytic therapy to prevent congestive heart failure after AMI.

**Methods:**

Twenty-six patients with successful PCI for acute ST-segment elevation anterior wall myocardial infarction were assigned to either a control group (*n* = 12) or a bone marrow mesenchymal stem cells (BM-MSC) group (*n* = 14). The control group received optimum post-infarction treatment, and the BMSC group received intracoronary delivery of autologous BMSC at 1 month after PCI with the optimum medical treatment. The primary endpoint was a left ventricular ejection fraction (LVEF) change from baseline to 4-month follow-up, as determined via myocardial single-photon emission computed tomography (SPECT).

**Results:**

The global LVEF at baseline (determined 3.5 ± 1.5 days after PCI) was 35.4 ± 3.0% in the control group and 33.6 ± 4.7% in the BM-MSC group. BMSC transfer enhanced left ventricular systolic function primarily in anterior wall myocardial segments adjacent to the LAD infarcted area. Four months later, via SPECT, global LVEF had increased by 4.8 ± 1.9% in the control group and 8.8 ± 2.9% in the BM-MSC group (*p* = 0.031). The cell transfer did not increase the risk of adverse clinical events, in-stent restenosis, or proarrhythmic effects. The echocardiographic evaluation also revealed a significant increase in the LVEF value from baseline to the 4-month (9.0 ± 4.7 and 5.3 ± 2.6%, *p* = 0.023) and 12-month (9.9 ± 5.2% and 6.5 ± 2.7%, *p* = 0.048) follow-up in the BM-MSC group but not in the control group.

**Conclusions:**

Intracoronary administration of autologous BM-MSC was tolerable and safe with significant improvement in LVEF at 4-month (SPECT and echocardiography result) and 12-month (echocardiography result only) follow-up in patients with anterior AMI.

## Introduction

Rapid reperfusion of the infarct-related coronary artery is of great importance to salvage ischemic myocardium and limit the infarct size in patients with acute myocardial infarction (AMI). Although percutaneous transluminal coronary angioplasty with stent implantation is the method of choice to re-establish coronary flow [1], delayed treatment leads to subsequent loss of cardiomyocyte and heart failure, which is a major cause of long-term morbidity and mortality. The loss of viable myocardium initiates an adverse process of left ventricular remodelling, leading to chamber dilatation and contractile dysfunction in many patients. In this respect, stem cell therapy has emerged as a novel alternative to repair damaged myocardium.

Many experimental studies have shown that cardiac transfer of un-fractionated bone marrow cells or mesenchymal stem cells (MSCs) and progenitor cells derived from bone marrow can enhance functional recovery after AMI [2, 3]. As such, stem cells and progenitor cells derived from bone marrow have been proposed for use to repair cardiac tissue after AMI in patients [4–6]. Early clinical investigations have indicated the feasibility of infusing autologous bone marrow cells into infarct-related coronary artery after an AMI [7–9]. Stem cell therapy for AMI has been reported to be safe in clinical trials conducted over the last several decades, but the therapeutic effects for AMI have not been presented with certainty using clinical data to conduct reviews and meta-analyses [10]. Further clinical evidence may require an increase in patient accessibility to advanced stem cell regenerative therapy, particularly this one using MSC. Results from clinical data [11] and from pre-clinical studies [12] have indicated the possibility of improving the therapeutic effect by using bone marrow-derived mesenchymal stem cells (BM-MSC) rather than un-fractionated bone marrow cells to treat AMI.

BM-MSCs can differentiate into multiple types of cells including cardiac muscle, and BM-MSCs are known to secret several kinds of cytokine and/or growth factors that allow the regeneration of the tissue micro-environment, including immune-modulation, cell death inhibition, and angiogenesis. Thus, the working mechanism of BM-MSCs benefitting AMI may allow for the regeneration of cardiac muscle (direct effect) and/or tissue micro-environment (paracrine effect). The manufacturer has qualified the potency of Cellgram-AMI®, the BM-MSCs injected into the patients in this study, to differentiate into cardiac muscle and produce paracrine cytokines and growth factors.

We tried to assess the safety and efficacy of intracoronary infusion of autologous BM-MSCs at 1 month after percutaneous coronary intervention (PCI) in the patients with anterior wall myocardial infarction. The study goal is to investigate long-term effects (up to 2 years) of BM-MSC treatment in AMI patients. This report presents interim data collected at 4 and 12 months that show improvements in the left ventricular function (LVEF) and discusses future expectations. Our data may add significant clinical evidence for the effect of BM-MSC therapy on AMI patients.

## Materials and Methods

### Study Design and Patient Criteria

The clinical study protocol was approved by the Institutional Review Board of the St. Carollo Hospital in 2011 (IRB SCH2011-006). Enrolled eligible patients signed an informed consent form and participated in the study at the St. Carollo Hospital from January 2012 to May 2015. Patients were eligible if they (i) were admitted to the hospital less than 24 h after the onset of chest pain; (ii) presented an electrocardiography (ECG) showing ST-segment elevation greater than 1 mm in two consecutive leads, greater than 2 mm in the precordial leads; and (iii) could be enrolled in the study less than 72 h after successful revascularization of anterior AMI (defined as residual stenosis less than 30% of left anterior descending artery [LAD] infarction) and EF ≤ 40%. We excluded patients with non-LAD infarction, cardiogenic shock, life-threatening arrhythmia, advanced renal or hepatic dysfunction, history of previous coronary artery bypass graft, history of hematologic disease and malignancy, major bleeding requiring blood transfusion, stroke or transient ischemic attack in the previous 6 months, use of corticosteroids or antibiotics during the previous month, major surgical procedure in the previous 3 months, cardiopulmonary resuscitation for more than 10 min within the previous 2 weeks, positive results for viral markers [human immunodeficiency virus (HIV), hepatitis B virus (HBV), hepatitis C virus (HCV), and Venereal Disease Research Laboratory (VDRL) test], and pregnancy or possible pregnancy.

Patients were assigned to either a control group (*n* = 12) or a BM-MSC group (*n* = 14). The control group received optimal post-infarction medical treatment, and the BM-MSC group received optimal medical treatment with intracoronary transfer of autologous BM-MSCs 30 ± 1.3 days after PCI. Because of ethical considerations, we decided not to conduct bone marrow aspiration and a sham left-heart catheterization in patients randomized to the control group.

All patients were required to have successful revascularization of a culprit lesion of LAD on coronary angiography at the time of randomization. All patients received aspirin (300 mg loading dose, then 100 mg daily) and clopidogrel (600 mg loading dose, then 75 mg daily) with optimal medical therapy according to the American College of Cardiology (ACC)/American Heart Association (AHA) guidelines for treatment of ST-segment elevation myocardial infarction (STEMI) [13–15]. The optimal medical therapy, including aspirin, clopidogrel, beta blocker, angiotensin-converting enzyme (ACE) inhibitor (or angiotensin receptor blocker), and statin, continued unless the drugs were contraindicated. The use of aspiration thrombectomy or a glycoprotein IIb/IIIa inhibitor during PCI was done as needed. All patients had successful PCI.

### Preparation of Autologous BM-MSC

After baseline myocardium SPECT, 20 to 25 mL of BM aspirates were obtained under local anesthesia from the posterior iliac crest at 3.0 ± 1.5 days after admission. All manufacturing and product testing procedures to generate clinical-grade autologous BM-MSC (Cellgram®-AMI) were carried out following good manufacturing practice (GMP) at Pharmicell Co., Ltd., Seongnam, Korea. The manufacturing practice has been described elsewhere [7], and the manufacturer’s standard operating procedure includes conducting quality assurance, quality control, and characterization of the clinical-grade autologous BM-MSCs.

### Cell Injection

The injection route of the BM-MSCs has been described elsewhere [16]. The packed final product of clinical-grade autologous BM-MSC (7.2 ± 0.90 × 107 cells) was gently transferred into the infusion syringe and mixed to minimize cell aggregation before infusion into the LAD via the central lumen of an over-the-wire balloon catheter (Maverick®, Boston Scientific, Natick, MA, USA). To allow the maximum contact time of the BM-MSC with micro-circulation of the LAD infarction territory, the balloon was inflated inside the stent at a low pressure to transiently interrupt antegrade blood flow during the infusions. The entire cell injection was done during three transient occlusions, each lasting 2 to 3 min. Between occlusions, the coronary artery was re-perfused for 3 min. After cell injection, close observation identified clinical changes and/or possible complications. Cardiac enzyme and electrocardiography measurements were repeated to assess periprocedural myocardial infarction (MI). The mean duration of autologous BM-MSC culture from BM aspiration to intracoronary injection was 25.0 ± 2.4 days.

### Follow-Up Visit and Endpoints

The study visits were scheduled at 1 and 4 months after PCI for the clinical and functional evaluation. Electrocardiogram-gated single-photon emission computed tomography (SPECT) was carried out at baseline (within 3–5 days after PCI) and 4 months (3 months after BM-MSC injection). Echocardiography was conducted to measure LVEF at 4 and 12 months after baseline observation. The primary endpoint of the study included absolute changes in the global LVEF from baseline to 4 months after PCI via SPECT. Echocardiography alone revealed altered global LVEF at 12 months. The secondary endpoints were changes in the left ventricular end-diastolic volume (LVEDV), left ventricular end-systolic volume (LVESV), and major adverse cardiac events (MACE). MACE was defined as the composites of any cause of death, myocardial infarction, revascularization of the target vessel, rehospitalization for heart failure, and life-threatening arrhythmia. MI was defined following the consensus statement of the Joint European Society of Cardiology (ESC)/American College of Cardiology (ACC)/American Heart Association (AHA)/World Heart Federation (WHF) Task Force for the Redefinition of Myocardial Infarction for clinical trials on coronary intervention [17]. Hence, periprocedural MI was defined as the levels of cardiac biomarkers (troponin or creatine kinase-MB [CK-MB]) > 3 times the 99th percentile of upper limit of normal (ULN) in patients with normal baseline levels and as a subsequent elevation > 3 times in CK-MB or troponin in patients with raised baseline levels. Target vessel revascularization (TVR) included bypass surgery or repeat PCI of the target vessel(s).

### Assessment of Left Ventricular Function

SPECT was used for the non-invasive measurement of LVEF. A single dose of technetium (99mTc) sestamibi prepared using the Cardiolite® kit (Dupont Merck Pharmaceutical Company, Billerica, MA, USA) was injected intravenously at rest, and data acquisition started 30–60 min later. SPECT data were acquired with a dual-headed gamma camera (Infinia H3000WT; GE Medical System, Tel Aviv, Israel) equipped with a low-energy, high-resolution collimator. Sixty-four images were obtained over a 180° orbit using 90° between the heads. Acquisitions were attenuation-corrected and gated for 16 frames/cardiac cycle. The total acquisition time was 20 min. Vendor-specific, computer-enhanced edge detection methods were used to assess the left ventricular (LV) epicardial and endocardial margins during the entire cardiac cycle. The computer calculated resting global LVEF from the gated SPECT images using an automated algorithm [18]. An analysis of the SPECT images was performed using two blinded independent investigators. Regional and global LV functions were measured using two-dimensional echocardiography according to the recommendations of the American Society of Echocardiography [19]. LVEF was measured from the end-diastolic and end-systolic volumes calculated by the Simpson method from two orthogonal apical views. Off-line assessment of all echocardiographic images was performed by one blinded independent investigator.

### Statistical Analyses

The primary endpoint was a change from baseline in the global LVEF at 4 months of follow-up. Improvement in global LVEF at 12-month follow-up evaluation was also observed. The secondary endpoints included changes in LVEDV and LVESV. ANOVA was used to compare the global LVEF changes in the two study groups and LVEF at baseline as a covariate. To estimate the treatment effect, differences in least-squares means and corresponding 95% CI were calculated based on the ANOVA model. We analyzed secondary endpoints using the same methods. The consistency of the reatment effect on the change in global LVEF was assessed across several subgroups. All statistical tests were two-sided with a significance level of *p* < 0.05. The homogeneity of the treatment groups at baseline was assessed using Student’s *t* test for continuous variables, and no marked deviations were shown from the normal distribution. Continuous variables are presented as mean ± standard deviation. Categorical data are presented as frequencies and percentages. Comparisons of continuous variables at baseline with those at follow-up were done with the paired *t* test. Comparison of non-parametric data between groups was undertaken using the Wilcoxon rank sum test and the Mann-Whitney test. Statistical significance was set to *p* < 0.05. Data were analyzed using SPSS for Windows ver. 15 (SPSS, Chicago, IL, USA).

## Results

### Characterization of Therapeutic Autologous BM-MSC

**Fig. 1:**
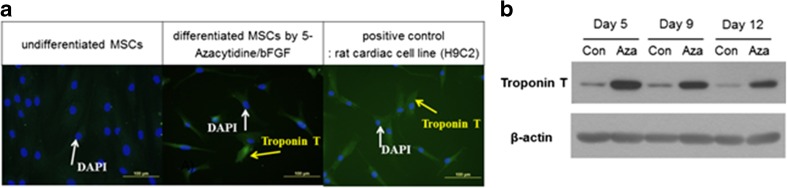
Differentiation of bone marrow mesenchymal stem cells to cardiac muscle cells. **a** Immunohistochemical analysis of the expression of troponin T, a cardiac muscle-specific marker in the 9-day cultured MSCs. Blue staining; 4′,6-diamidino-2-phenylindole(DAPI)/green staining; troponin T. **b** Western blot analysis of 5-azacytidine/bFGF-induced cardiac muscle-specific protein-troponin T expression

**Fig. 2:**
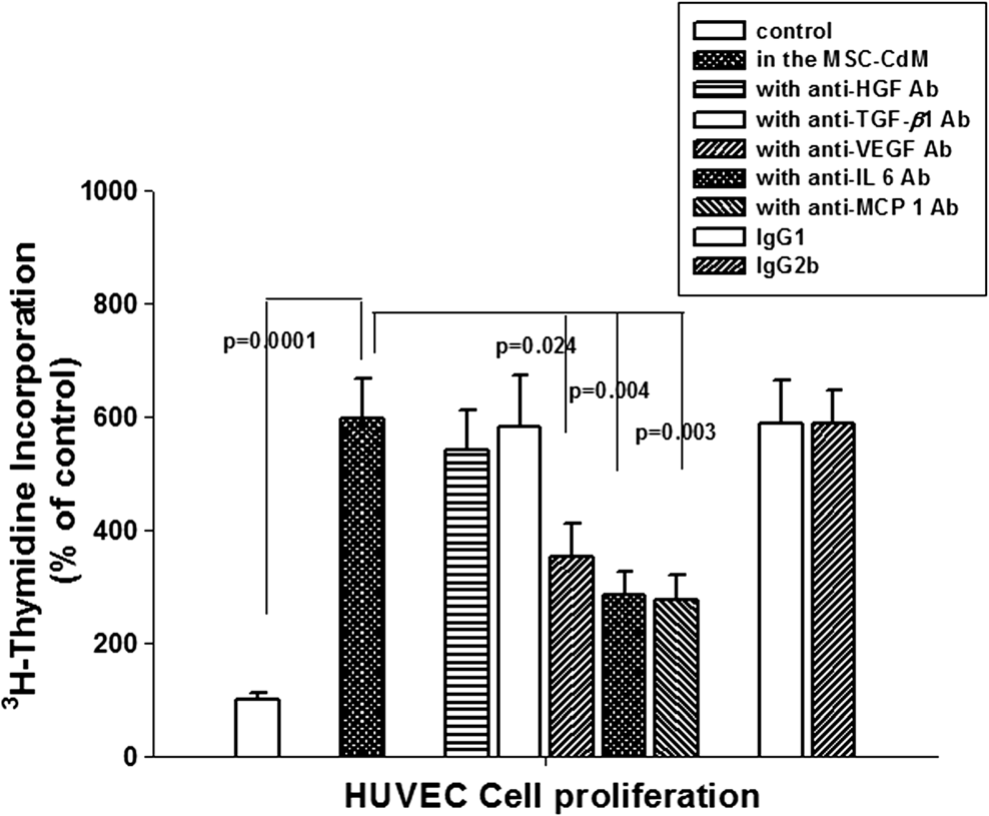
Angiogenesis-inducing factors secreted by BM-MSCs: role of defined cytokines on the angiogenesis were analyzed by measuring the HUVEC (vascular endothelial cell line) cell proliferation in the MSC culture media (MSC-CdM) with or without neutralizing antibodies (5 μg/mL). Effect of neutralizing antibody isotypes were analyzed concurrently: IgG1 (for αTGF-b, αHGF, and αIL-6) and IgG2b (for αVEGF, αMCP-1). MSC-CdM was obtained from three different culture lots

The characteristics and quality of the therapeutic autologous BM-MSC were assured by the manufacturer (Pharmicell Co., Ltd) adhering to GMP. The International Society of Cell & Gene Therapy (ISCT) standard (Table 1) [20] was used to characterize the therapeutic MSCs (data not shown). The potency of therapeutic MSCs was determined at the manufacturer’s GMP facility by observing the differentiation ability to cardiac muscle and secretion of related cytokines/growth factors. The ability to differentiate into cardiac muscle was determined by measuring the expression of troponin T on the 5-azacytidine/basic fibroblast growth factor (bFGF) induced differentiated MSCs (Table 2 and Fig. 1). The pleiotropic mechanism of therapeutic MSCs was assayed by measuring the angiogenic effect of MSC-secreted factors including vascular endothelial growth factor (VEGF), interleukin-6 (IL-6), and monocyte chemoattractant protein-1 (MCP-1) (Fig. 2). MSC culture media induced human vascular endothelial cell (HUVEC) proliferation was reduced by the neutralizing antibodies against VEGF, IL-6, MCP-1 but not by hepatocyte growth factor (HGF) or transforming growth factor-beta (TGF-β). Isotype of neutralizing antibodies (IgG1 for HGF or TGF-β and IL-6: IgG2b for VEGF and MCP-1) has no toxic effects on the proliferation of HUVEC (Fig. 2). Only autologous MSCs that passed quality assurance and quality control tests—including sterility, mycoplasma, endotoxin, and virus contamination (data not shown) tests—were delivered as a therapeutic cells.

**Table 1:** Therapeutic MSC characteristics (MSC identification criteria by “International Society of Cellular Therapy” Cytotherapy 2006)

1	Adherence to plastic in standard culture conditions		
2	Phenotype	Positive (≥ 95% +)	Negative (≤ 2% +)
		CD105	CD45
		CD73	CD34
		CD90	CD14 or CD11b
			CD79a or CD19
			HLA-DR
3	In vitro differentiation	Osteoblasts, adipocytes, chondroblasts

**Table 2:** Typical expression of cardiac muscle cell-specific markers on the therapeutic MSCs: immunohistochemistry

	Expression of cardiac muscle cell-specific markers						
	α-sarcomeric actin	Troponin I	Troponin T	MHC	MRLC	GATA-4	Nkx 2.5
Undifferentiated MSCs	+++	++	±	±	±	+++	±
MSC differentiated with 5-azacytidine /bFGF	+++	+++	+/++	+	+/++	+++	+/++

### Clinical Characteristics

Between January 2012 and May 2015, 30 patients provided informed consent to participate in this trial. Twenty-six out of 30 patients were randomly allocated to each treatment group. Four patients were withdrawn because they could not undergo M-SPECT. The final randomized cohort included 12 patients in the optimal post-infarction treatment group (control group) and 14 patients in the autologous BM-MSC and optimal post-infarction treatment group (BM-MSC group). The baseline patient characteristics for both groups were well matched (Table 3). No differences were found in the two groups with respect to cardiovascular risk factors and medical treatments. The Killip class and coronary angiographic characteristics, including the only LAD infarction patient, were also similar between the two groups. Primary PCI was carried out in all cases, and there were no significant differences in procedural characteristics and time intervals from chest pain onset to treatment. All patients received optimal post-infarction medical treatment (Tables 3 and 4).

**Table 3:** Baseline characteristics of the patients and concomitant therapy

Characteristics	Control group (*n* = 12)	MSC group (*n* = 14)	*p* value
Age (year)	57.8 ± 8.9	55.3 ± 8.6	0.479
Male sex—no (%)	12 (100)	14 (100)	
Risk factors			
Hypertension (%) Diabetes mellitus (%) Hyperlipidemia (%) Smoking (current or former) (%) Family history of coronary heart disease (%)	5 (41.7) 2 (16.7) 0 (0) 5 (41.7) 1 (8.3)	5 (35.7) 3 (21.4) 2 (14.3) 5 (35.7) 3 (21.4)	0.756 0.759 0.173 0.756 0.356
Killip class (%)			
Killip I Killip II	10 (83.3) 2 (16.7)	13 (92.9) 1 (7.1)	0.449 0.580
Coronary artery disease (%)			
1 vessel 2 vessel 3 vessel	8 (66.7) 3 (25.0) 1 (8.3)	11(78.6) 2(14.3) 1(7.1)	0.495 0.490 0.910
PCI for additional stenoses in non-infarct-related vessels (%)	0 (0)	1(7.1)	0.345
Time from symptom onset to first reperfusion therapy (min)	257.7 ± 303.2	245.1 ± 331.4	0.921
Medication (%)			
Aspirin	12 (100)	14 (100)	
Clopidogrel Ticagrelor Beta blocker ACEi or ARB	6 (50.0) 2 (16.7) 12 (100) 10 (83.3)	6 (42.9) 5 (35.7) 13 (92.9) 13 (92.9)	0.716 0.275 0.345 0.449
Vital signs			
Initial systolic BP (mmHg) Initial diastolic BP (mmHg) Initial pulse rate (beat per min)	128.5 ± 20.8 77.8 ± 12.8 80.2 ± 10.6	138.6 ± 28.0 82.1 ± 15.8 75.3 ± 11.3	0.315 0.448 0.270

Values are expressed as mean ± SD or number of patients (%)

*MSC* mesenchymal stem cell, *LAD* left anterior descending artery, *ACEi* angiotensin-converting enzyme inhibitor, *ARB* angiotensin receptor blocker, *BP* blood pressure

**Table 4:** Time intervals from symptom to treatment

Interval	Control group (*n* = 12)	MSC group (*n* = 14)	*p* value
Symptom to door time (h)			
≤ 2 2–6 > 6	8 (66.7) 2 (16.7) 2 (16.7)	10 (71.4) 3 (21.4) 1 (7.1)	0.793 0.759 0.449
Symptom to balloon time (h)			
≤ 2 2–6 > 6	4 (33.3) 6 (50.0) 2 (16.7)	4 (28.6) 9 (64.3) 1 (7.1)	0.793 0.462 0.449
Symptom to initial SPECT (days)	3.8 ± 1.3	2.8 ± 2.6	0.256
Symptom to follow-up SPECT (days)	145.9 ± 207.7	119.4 ± 55.6	0.649
Symptom to initial Echo (days)	1.2 ± 0.9	1.6 ± 0.8	0.235
Symptom to follow-up Echo (days)	94.5 ± 46.1	92.7 ± 42.0	0.927

*SPECT* single-photon emission computed tomography, *Echo* echocardiography

### Quantitative Analyses of LV Function by SPECT

**Fig. 3:**
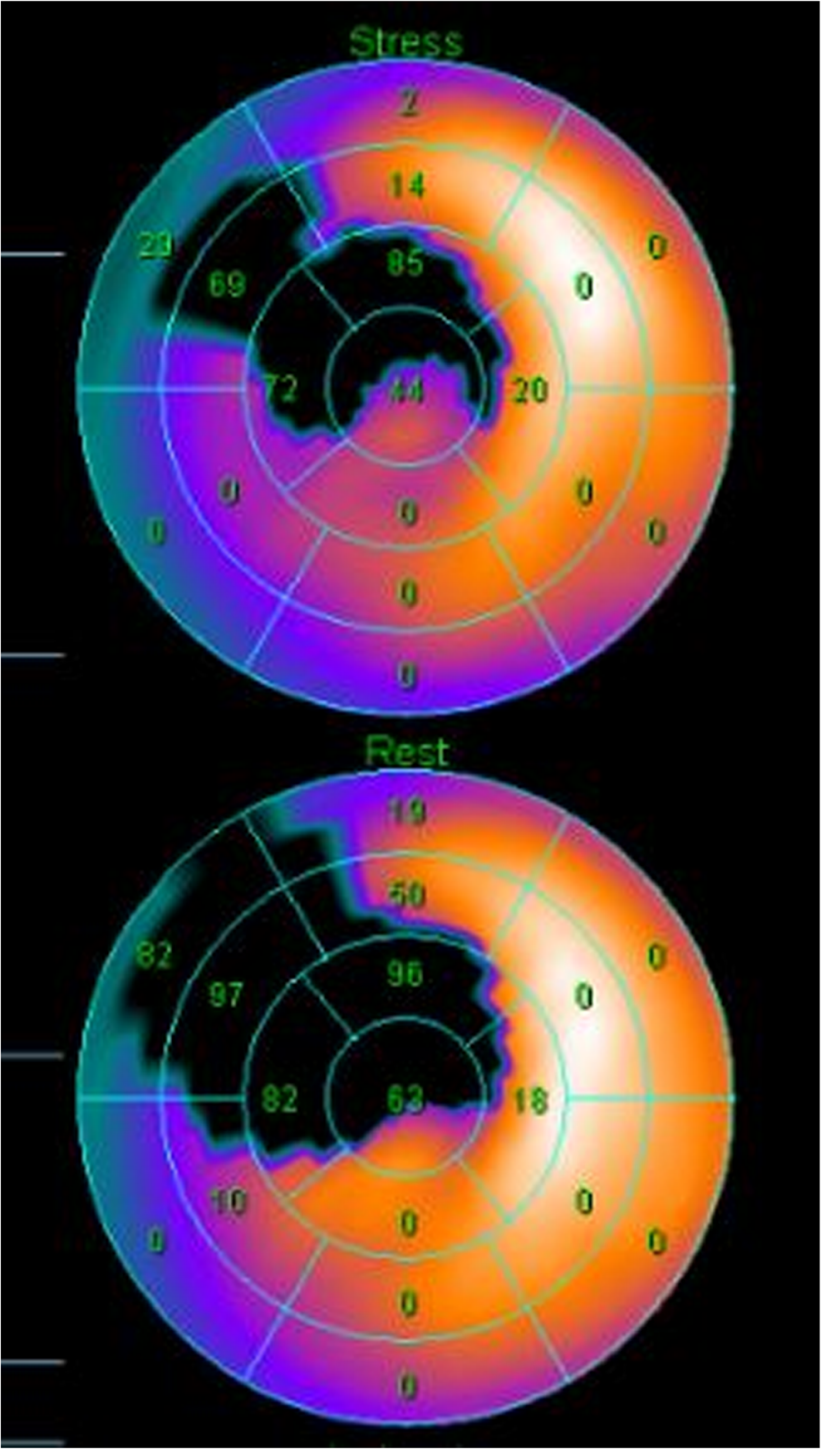
Representative color-coded images showing systolic wall motion at baseline and 4 months follow-up in bone-marrow mesenchymal stem cells patient that had an anterior acute myocardial infarction, Bright colors indicate good systolic wall motion, whereas dark colors indicate poor wall motion. Note improved functional recovery in this patient

**Fig. 4:**
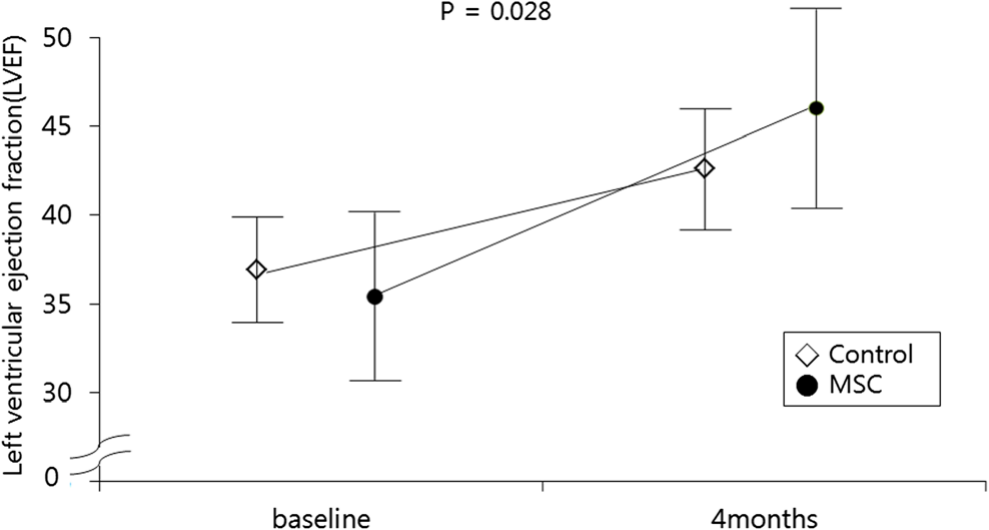
Changes of LVEF by SPECT at baseline and 4 months after MSC delivery. LVEF, left ventricular ejection fraction; SPECT, single-photon emission computed tomography

**Fig. 5:**
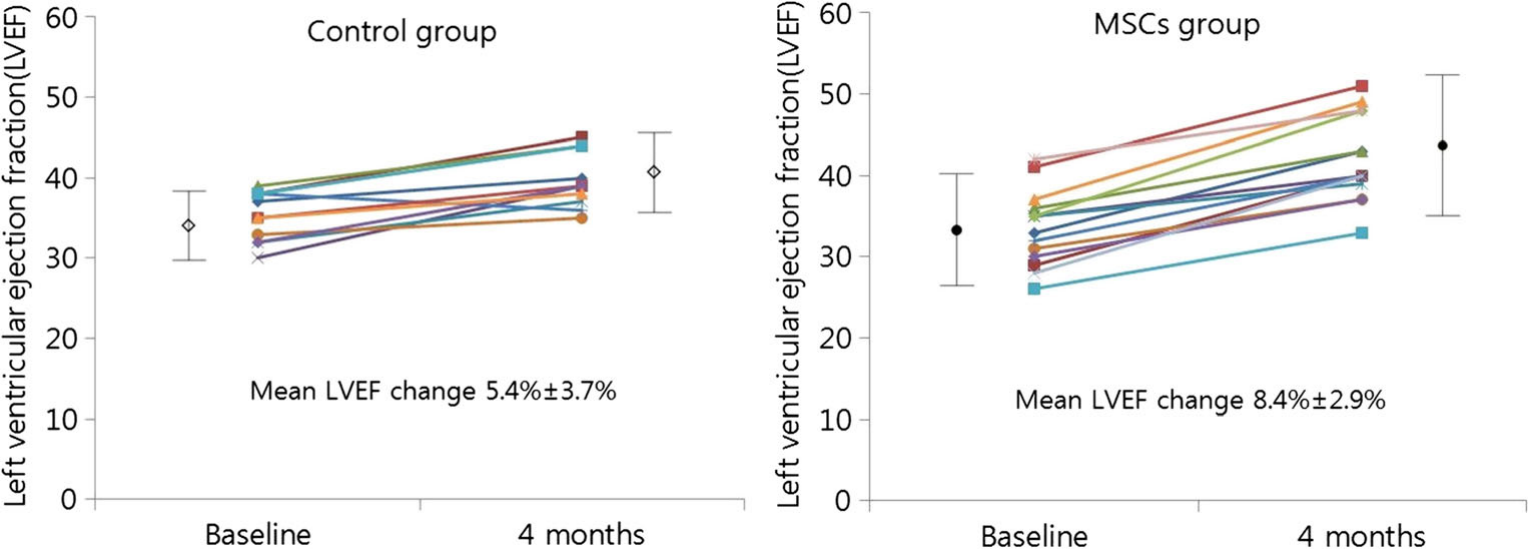
Impact of MSCs treatment on LVEF by SPECT and echocardiography at 4- and 12-month after PCI. MSCs, mesenchymal stem cells; LVEF, left ventricular ejection fraction; SPECT, single-photon emission computed tomography

Baseline LVEF was similar between the two groups (35.4 ± 3.0% in the control group and 34.2 ± 4.7% in the BM-MSC group, *p* = 0.251) (Table 5). The absolute change in the global LVEF from baseline to 4 months improved significantly in the BM-MSC group compared to the control group (8.8 ± 2.9 vs. 4.8 ± 1.9%, *p* = 0.031) (Table 5 and Figs. 3, 4, and 5). Baseline and 4-month LVEDV and LVESV showed no significant differences. The changes in LVEDV and LVESV also did not differ significantly at the 4-month follow-up in either group. Representative color-coded images showing the effects of the BM-MSC transfer on left ventricular function are shown in Fig. 3.

**Table 5:** Quantitative measures of left ventricular function by SPECT and echocardiography

Measurements	Control group (*n* = 12)	MSC group (*n* = 14)	*p* value
SPECT			
Global LVEF (%)			
Baseline 4 months	35.4 ± 3.0 39.8 ± 3.3	34.2 ± 4.7 42.7 ± 5.9	0.251 0.046
LVEDV (mL)			
Baseline 4 months	140.4 ± 19.2 133.2 ± 16.2	144.6 ± 31.5 135.9 ± 38.7	0.690 0.825
LVESV (mL)			
Baseline 4 months	92.8 ± 14.8 79.3 ± 13.4	96.1 ± 22.9 81.9 ± 28.1	0.671 0.766
Echocardiography			
Global LVEF (%)			
Baseline 4 months 12 months	37.4 ± 1.7 42.0 ± 2.6 44.5 ± 2.3	35.1 ± 4.5 44.1 ± 5.8 45.0 ± 4.2	0.116 0.255 0.124
LVEDV (mL)			
Baseline 4 months 12 months	102.3 ± 21.2 98.8 ± 20.3 101.3 ± 18.0	112.1 ± 41.8 103.7 ± 28.2 104.4 ± 28.6	0.346 0.117 0.745
LVESV (mL)			
Baseline 4 months 12 months	61.6 ± 11.3 54.1 ± 8.4 61.1 ± 12.3	67.8 ± 27.2 59.6 ± 18.4 60.4 ± 18.1	0.446 0.098 0.913
Changes at 4 months			
SPECT			
LVEF (%) LVEDV (mL) LVESV (mL)	4.8 ± 1.9 7.2 ± 4.1 13.6 ± 7.3	8.8 ± 2.9 8.7 ± 24.9 14.2 ± 18.2	0.031 0.822 0.907
Echocardiography			
LVEF (%) LVEDV (mL) LVESV (mL)	5.3 ± 2.6 8.5 ± 30.3 10.5 ± 16.9	9.0 ± 4.7 5.5 ± 32.0 7.2 ± 18.4	0.023 0.806 0.641
Change at 12 months			
Echocardiography			
LVEF (%) LVEDV (mL)	6.5 ± 2.7 1.0 ± 13.4	9.9 ± 5.2 7.7 ± 19.6	0.048 0.385
LVESV (mL)	0.5 ± 16.0	7.4 ± 22.0	0.377

Values are expressed as mean ± SD or number of patients (%)

*MSC* mesenchymal stem cell, *SPECT* single-photon emission computed tomography, *LVEF* left ventricular ejection fraction, *LVEDV* left ventricular end-diastolic volume, *LVESV* left ventricular end-systolic volume

### LV Function as Revealed by Echocardiography

Echocardiographic observation also indicated a similar baseline LVEF level in the control group and the BM-MSC group (37.4 ± 1.7 and 35.1 ± 4.5%, respectively, *p* = 0.116). The echocardiographic evaluation also revealed a significant increase in LVEF from baseline until the 4 months (9.0 ± 4.7 and 5.3 ± 2.6%, *p* = 0.023) and 12 months (9.9% ± 5.2 and 6.5% ± 2.7%, *p* = 0.048) of follow-up in the BM-MSC group but not in the control group (Table 5). Volumetric analyses of LV end-diastole and end-systole at baseline and at 4- and 12-month follow-up showed no significant differences between the two groups.

### Safety and Clinical Outcomes

All procedures related to the BM aspiration and MSC transplantation were well tolerated. There were no serious inflammatory reactions or bleeding complications at the iliac puncture site after BM aspiration. Patients had no or mild angina during balloon inflation for infusion of BM-MSC. There were no serious procedural complications related to intracoronary administration of the BM-MSC, such as ventricular arrhythmias, thrombus formation, or dissection. Periprocedural MI did not occur in all patients. There were no deaths, *rehospitalization*, MI, TVR, stent thrombosis, life-threatening arrhythmia, or stroke in both groups during the 4- and 12-month follow-up period. Except for a few premature ventricular beats in both groups, no significant arrhythmic events were recorded on 24-h ECG monitoring (Table 6). No significant changes were seen in LVEDV, LVESV as secondary endpoints, and MACE.

**Table 6:** Clinical events during follow-up

Event	MSC group (*n* = 14)	Control group (*n* = 12)	*p* value
Event before hospital discharge			
Death	0	0	
Myocardial infarction	0	0	
4-month follow-up (cumulative)			
Death	0	0	
Myocardial infarction	0	0	
Rehospitalization for heart failure	0	0	
Revascularization			
Target vessel revascularization	0	0	
Stent thrombosis	0	0	
Non-target vessel revascularization	0	0	
Cerebral infarction	0	0	
Documented			
≥Bigeminic ventricular premature beat	1	2	0.867
Ventricular arrhythmia with syncope	0	0	
Atrial fibrillation	1	0	0.345
12-month follow-up (cumulative)			
Death	0	0	
Myocardial infarction	0	0	
Rehospitalization for heart failure	0	0	
Revascularization			
Target vessel revascularization	0	0	
Stent thrombosis	0	0	
Non-target vessel revascularization	0	0	
Cerebral infarction	0	0	
Documented			
≥Bigeminic ventricular premature beat	0	1	0.271
Ventricular arrhythmia with syncope	0	0	
Atrial fibrillation	0	0	

## Discussion

Our study addresses the effect of the BM-MSC on left ventricular functional recovery after acute anterior STEMI with left ventricular dysfunction. We observed that the infusion of BM-MSC into the infarct-related coronary artery of LAD significantly improved the recovery of global LVEF 3 months after the BM-MSC injection, 4 months after the PCI. The improvement in LVEF was continuously observed at 12 months of follow-up. The BM-MSC infusion was tolerable without serious complications. The improvement in the global LVEF in the treatment group was mostly due to improved regional systolic wall motion in the infarct border zone. Left ventricular end-diastolic volumes did not decrease, indicating that BM-MSC transfer did not improve left ventricular remodeling. Several research and clinical reports [21–24] have suggested that the main mechanism for the improvement of LV contractile function in AMI patients is neovascularization induced by intracoronary infusion of bone marrow-derived cells (BMC). This may explain the LVEF improvement without volumetric change in the BMC-MSC treated group observed in this study. BMC-MSC (Cellgram®-AMI) used in this study has been confirmed to be able to induce angiogenesis through secretion of VEGF, IL-6, and MCP-1 (Fig. 2).

Longer follow-up may be required to assess the impact of MSCs into the LAD coronary artery on long-term left ventricular structural adaptation after AMI. A previous 6-month follow-up report of clinical responses evaluating autologous MSCs (Cellgram®-AMI) in AMI patients showed a primary endpoint of ≥ 4.3% improvement in LVEF compared to the control group, based on SPECT data [7]. Similar clinical responses were observed in this study based on SPECT data at the 4-month follow-up evaluation (≥ 4.0% (8.8 ± 2.9 minus 4.8 ± 1.9%), suggesting improved LVEF in the BM-MSC-treated group compared to the control group (*p* = 0.031).

Clinical studies for stem cell therapy for AMI have been reported for decades, evidencing the safety of the cell therapy. Pre-clinical studies have shown MSCs are better than bone marrow progenitor cells to treat myocardial infarction [12]. However, most studies have been carried out with bone marrow-derived mononuclear cells, and the clinical response was defined as moderately to not clinically relevant. A review in the Cochrane library (“Stem cell treatment for acute myocardial infarction,” Fisher et al., 2015) [11] indicated that fewer than 10% of the studies dealt with MSCs (4 out of 41 individual studies). Thus, even the clinical outcomes when compared to those of the no-cell group (surrogate response measured to improve LVEF by SPECT at less than 12 months) reveal better result with BM-MSC (LVEF ≥ 4.3 vs 2.56% for 1 MSC study; Lee et al. 2014 [7] vs. 6 bone marrow mononuclear cell study combined) [11], so more studies should obtain further evidence (Curr Treat Options Cardiovasc Med. 2014) [10]. Our results also present an encouraging clinical effect of intracoronary infused autologous BM-MSC that met the primary endpoint of an improvement in LVEF of **~** 4.0% in the BM-MSC-treated group compared to control group at 4 months by SPECT.

The safety of intracoronary administration to deliver stem cells including MSCs has been accepted since Strauer et al. [25, 26] introduced the method. The therapeutic importance of recruiting MSCs into the infarcted myocardium [27, 28] has been noted, and ischemic preconditioning induced by transient balloon occlusion may have positive role for this phenomenon.

A consensus has been reached regarding research and clinical applications of the therapeutic mechanism of MSCs. Both target tissue regeneration and the pleiotropic effect of secretome (Cell Stem Cell, 2012) [29] are considered as important mechanisms of action, and the potency of therapeutic BM-MSCs used in this study (Cellgram®-AMI) was measured in both ways. The manufacturer has proven that the MSCs could be differentiated into cardiac muscle and that secretomes including VEGF, IL-6, and MCP-1-induced angiogenesis, which may be a major benefit for ischemic tissue.

Although encouraging clinical data was obtained, this study has several limitations, including the small number of participants, the relatively early time point for the data collection, and the limited means for LV functional measurement or endpoint measurement. However, in this report, only interim data is presented with LVEF improvement. A larger scale and a long-term follow-up study representing “post-marketing surveillance (PMS) of Cellgram®-AMI” was performed to define clinical endpoints including incidence of heart failure and survival along with an increase in global LVEF to elucidate the safety and therapeutic benefits of autologous MSC infusion in AMI patients. Data of a total of 100 patients were finally collected in February 2018 and analyzed. Results will be presented in a separate report. Based on preliminary analysis of PMS study, the cardiographic data of 78 patients, LVEF was significantly improved at 6 months after BM-MSC treatment (5.73 ± 0.79% in change from baseline LVEF 42.46 ± 10.23 to 48.19 ± 10.38% at 6 months, *p* < 0.0001) (data not shown).

In conclusion, intracoronary administration of autologous BM-MSC at 1 month after PCI is tolerable and safe with a significant improvement in LVEF at the 4- as well as 12-month follow-up in patients with acute anterior wall myocardial infarction.

## Abbreviations

AMI: Acute myocardial infarction
BM-MSC: Bone marrow-derived mesenchymal stem cells
LVEF: Left ventricular ejection fraction
LVEDV: Left ventricular end-diastolic volume
LVESV: Left ventricular end-systolic volume
PCI: Percutaneous coronary intervention
STEMI: ST-segment elevation myocardial infarction
MACE: Major adverse cardiac events
LAD: Left anterior descending artery
ISCT: International Society of Cell & Gene Therapy
VEGF: Vascular endothelial growth factor
IL-6: Interleukin-6
MCP-1: Monocyte chemoattractant protein-1
HGF: Hepatocyte growth factor
TGF-β: Transforming growth factor-beta
HUVEC: Human vascular endothelial cell
bFGF: Basic fibroblast growth factor
DAPI: 4′,6-Diamidino-2-phenylindole
